# Development of a Multi-technique Characterization Portfolio for Stainless Steels Exposed to Magnox Reprocessing Liquors

**DOI:** 10.1021/acsomega.3c07240

**Published:** 2023-11-17

**Authors:** Daniel N. T. Barton, Anna E. Denman, Tatiana Grebennikova, Thomas Carey, Dirk L. Engelberg, Clint A. Sharrad

**Affiliations:** † Department of Chemical Engineering, The University of Manchester, Oxford Road, Manchester M13 9PL, U.K.; ‡ Department of Earth and Environmental Sciences, The University of Manchester, Oxford Road, Manchester M13 9PL, U.K.; § National Nuclear Laboratory, Chadwick House, Warrington WA3 6AE, U.K.; ∥ Department of Materials, The University of Manchester, Oxford Road, Manchester M13 9PL, U.K.

## Abstract

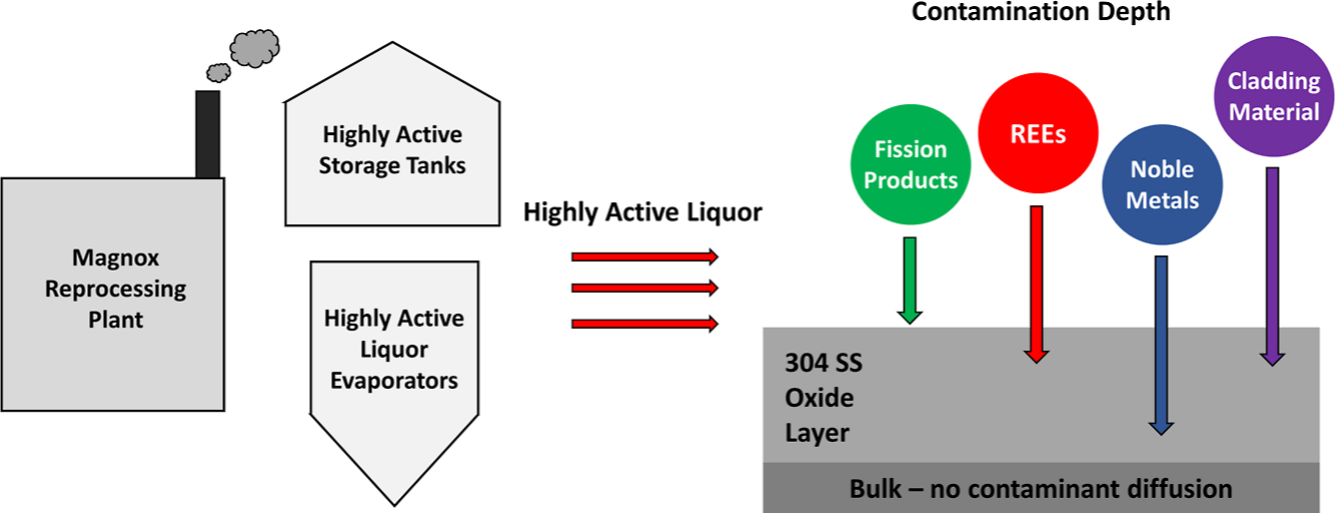

AISI Type 304 stainless steel coupons have been exposed to a simulant aqueous environment representative of the Magnox Reprocessing Plant (MRP) at Sellafield, UK. The experiments were performed for extended time periods (up to 420 days) at elevated temperatures to develop a comprehensive understanding of the extent, nature, and depth of contamination for pipework and vessels in Magnox spent nuclear fuel reprocessing environments. This will inform upcoming decommissioning work which represents a major post-operational challenge. Previous relevant literature has focused on developing fundamental understanding of contamination mechanisms of stainless steels in simplistic, single-element systems, which lack elements of industrial relevance. Contamination behavior is expected to be drastically different in these more complex environments. A characterization portfolio has been developed to enable detailed assessment of corrosion and contamination behavior in acidic reprocessing environments. Solution, surface, and depth analysis determined that uptake was dominated by the elements present in highest concentrations within the environment, namely, Mg, Nd, and Cs. Most contaminants were incorporated into a relatively thin surface oxide layer (<100 nm) in metal oxide form, although there were some exceptions (Cs and Sr). Grain boundary etching was present despite very low corrosion rates (3 μm year^–1^). As a result of this lack of corrosion, diffusion of contaminants beyond the immediate surface (10–20 nm) did not occur, evidenced through depth profiling. As a result of these findings, surface-based decontamination techniques minimizing excess secondary waste generation can be further developed in order to reduce the environmental and economic burden associated with decommissioning activities.

## Introduction

The Magnox Reprocessing Plant (MRP) at Sellafield, UK, ceased operations in July 2022 after almost 60 years of reprocessing spent nuclear fuel from the UK’s Magnox reactor fleet.^1^ The MRP utilized an adapted Plutonium Uranium Reduction Extraction (PUREX) process, where the spent fuel was dissolved in nitric acid (after de-cladding), before the uranium and plutonium was separated by solvent extraction after addition of ferrous sulfamate.^2,3^ With the uranium and plutonium separated from the fission products and minor actinides, highly radioactive aqueous liquors were transferred to evaporators for aqueous waste volume reduction prior to storage and vitrification.^4^

The metallic pipework and vessels within the MRP were predominantly manufactured from niobium- and titanium-stabilized stainless steels, suffering from extensive intergranular corrosion after exposure to nitric acid.^5^ The metallic infrastructure having been subjected to complex and corrosive acidic liquors for several decades is expected to be extensively contaminated with a wide range of species, including fission products and fuel components. This infrastructure is likely to be categorized as Intermediate Level and Low Level Waste as a result of extended exposure to the radioactive liquors due to the uptake of alpha- and/or beta/gamma-emitting radionuclides to the surface. This infrastructure will require decontamination prior to hands-on dismantling operations to minimize radiation dose exposure.^6^

With a move to the decommissioning stage imminent, detailed characterization plans and procedures are required for the successful identification of the extent and nature of contamination on these metallic surfaces. This knowledge is required to underpin the optimization of Post Operational Clean Out (POCO) and decommissioning strategies which have the potential to achieve waste volume minimization. A key aim of The Nuclear Decommissioning Authority is to reduce secondary waste volumes by 70% by 2030.^7^ If this is achieved, this will reduce the economic and environmental burdens associated with waste management and disposal. The development of optimized decontamination processes is therefore a necessity, with effective decontamination potentially leading to waste recategorization, which in turn could result in significant cost savings per cubic meter of waste.^8^ Recycling and reuse of the stainless steel once full decontamination is complete could also become a possibility.

This study aimed to develop a comprehensive understanding of how stainless steel was corroded and contaminated by a complex aqueous simulant solution, replicating environments observed after spent fuel dissolution in the MRP. Some studies have similarly explored contamination of stainless steels with simulant aqueous solutions, either from reprocessing facilities or from reactor primary coolant circuits, but with shorter exposure times and lower temperatures.^9−11^ The simulant studies placed greater focus on contaminant speciation identification, presenting a possible opportunity for comparison in this study, despite the different experimental conditions. Other literature studies have focused solely on single-element contamination of stainless steel in non-competing systems,^12−19^ whilst other work suggests that more complex multi-element systems can behave very differently.^9^

Typically, immersion studies last no longer than 30 days, making predictions of long-term corrosion and contamination behavior challenging.^20^ Here, AISI Type 304 stainless steel, similar to the grades employed in the MRP, has been exposed to a Magnox simulant solution for up to 420 days, with the steel surfaces being analyzed by a set of techniques that are available to the industry, including scanning electron microscopy (SEM), X-ray photoelectron spectroscopy (XPS), and laser ablation—ICPMS. Solutions were analyzed with inductively coupled plasma–optical emission spectroscopy (ICP–OES) to understand contaminant uptake variation over time, up to 100 days of exposure. The findings of this work can be used to optimize post-operational processes and reduce the future waste burden associated with decommissioning and decontamination.

## Experimental Methods

### Materials and Sample Preparation

AISI Type 304 stainless steel obtained from Lakeland Steel Ltd. was utilized in this study as it is similar to the nitric acid grade (NAG) stainless steels utilized in the Magnox Reprocessing Facility at Sellafield.^5^ The steel was received with a 2B finish and was exposed in the as-received condition to mimic the same engineering surface as used in plant. 20 × 20 × 4 mm stainless steel coupons, corresponding to a total surface area of 11.2 cm^2^, were exposed to the acidic liquors for a period of up to 420 days. Prior to immersion studies, all steel coupons were cleaned ultrasonically for 10 min in both acetone and deionized water. Its elemental composition is found in Table 1.

**Table 1:** Elemental Composition by Weight Percentage (wt %) for As-Received 304 Stainless Steel

element	Cr	Ni	C	Mn	Si	P	S	Fe
wt %	18.2	8.3	0.063	1.18	0.412	0.065	0.004	balance

The acidic simulant liquor representative of a Magnox spent nuclear fuel reprocessing environment was synthesized with a composition as detailed in Table 2. All salts were used in the nitrate form (≥99% purity), other than samarium (oxide form, 99.9% purity). Salts were initially added to 250 mL of 4 M HNO_3_, with the total volume being made up to 1 L with deionized water to give a final acidity of 1 M, in line with aqueous liquor concentrations post-evaporation. The solution was then heated and stirred at 50 °C for 1 h to aid dissolution.

**Table 2:** Approximate Concentrations of the Elements Present in the Magnox Simulant Solution Used to Contaminate 304 Stainless Steel Coupons for up to 420 Days at 50 °C

element	concentration (g L^–1^)	element	concentration (g L^–1^)
Mg	3.6	Rh	0.065
Al	2.4	Pd	0.14
Cr	0.20	Cs	1.9
Fe	1.6	La	0.38
Ni	0.12	Ce	0.71
Sr	0.23	Pr	0.36
Y	0.15	Nd	1.22
Ru	0.20	Sm	0.14

The 304 stainless steel coupons were immersed in 50 mL of the Magnox solution and maintained at 50 °C for up to 420 days. 100 μL aliquots of solution were taken at regular intervals and diluted appropriately for analysis by ICP–OES. The procedure for coupon preparation for mass loss and corrosion rate measurements has been detailed in the authors’ previous study.^21^

### Characterization Techniques

A comprehensive analysis of the acidic liquor in contact with the 304 stainless steel coupons was undertaken with ICP–OES. Optical emission lines for all 16 elements present in solution were analyzed using an Analytik Jena PlasmaQuant PQ 9000 ICP–OES, in order to assess contaminant uptake onto the stainless steel coupons from the solution.

Contaminant uptake at time *t*, *q*_*t*_ (g m^–2^), was determined using eq 1, adapted from the standard equation for uptake capacity in ion-exchange studies
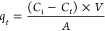
1where *C*_i_ and *C*_*t*_ are the solution concentrations initially and at time, *t* (g L^–1^), *V* is the volume of the solution (L), and *A* is the surface area of the entire coupon (m^2^).

SEM (FEI Quanta 250 FEG-SEM), XPS (Kratos Axis Ultra DLD XPS), and laser ablation–ICPMS (Analyte Excite+, Teledyne CETAC Technologies) were used for surface imaging, speciation analysis, and depth profiling, respectively. For a more detailed summary of the experimental parameters employed in this study, please refer to the authors’ previous work.^21^

## Results

### Long-Term Corrosion Behavior

Assessment of mass loss over time in acidic liquors can be used to predict corrosion rates that may be expected in similar environments on plant. Here, the final nitric acid concentration of the Magnox simulant was 1 M. The corrosion potential of low concentration nitric acid systems has been seen to lie well within the passive corrosion domain, even at high temperatures.^22,23^ The extent of corrosion was, therefore, not expected to be high. In fact, it was expected that passivation would be maintained throughout the entirety of the experiment.

However, the addition of some species has been shown to have a profound effect on mass loss acceleration.^24−26^ It was therefore important to confirm whether the wide range of elements in solution could contribute to an increase in the corrosion rate.

**Figure 1:**
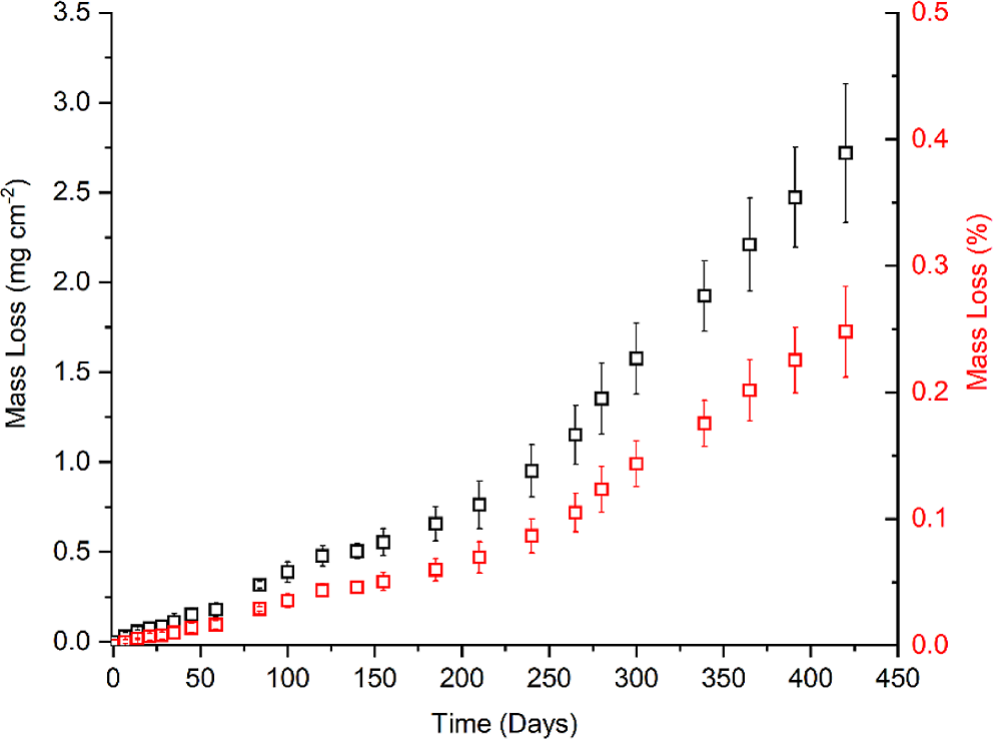
Variation in mass loss with respect to surface area (mg cm^–2^) represented by a black □ and variation in overall mass of the 304 stainless steel coupons (%) represented by a red □, after immersion in the Magnox simulant solution at 50 °C for 420 days.

Figure 1 shows that the mass loss (black) from the whole coupon was very small (∼3 mg cm^–2^), with the equivalent mass loss of the whole coupon being less than 0.3% (red). A combination of the low acid concentration along with the concentrations of dissolved species in the Magnox liquor appeared to have a minimal effect on elevating the corrosion rate.

**Figure 2:**
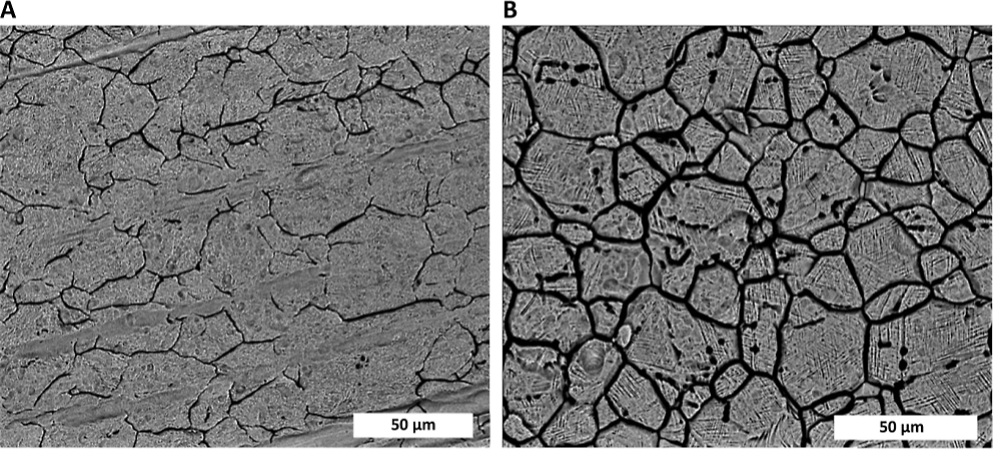
Secondary electron scanning electron micrographs of a 304 stainless steel coupon exposed to the Magnox simulant solution at 50 °C for 270 (A) and 420 (B) days.

After the coupons were exposed for 270 days, grain boundary etching was seen to become more widespread across the surface of the coupons (Figure 2A), although initial surface features (2B finish marks) were still visible. The increase in the rate of mass loss at approximately 270 days, as can be seen in Figure 1, may correspond to the onset of widespread grain boundary etching, resulting in a greater surface area available for corrosive attack.

As a result of long-term exposure of the coupons to the acidic media (420 days), clear, widespread grain boundary etching was seen (Figure 2B). There was also some evidence of grain dropping of small surface-based grains, but this was not prevalent. These corrosion features can provide pathways for contaminant diffusion into the bulk substrate, with the likelihood of this occurring being considerably higher if there is widespread grain dropping and intergranular attack, as can be seen with more concentrated acidic solutions.^21^

### Solution Analysis for Contaminant Uptake Determination

Steady-state contaminant uptake was achieved relatively quickly for most species (within 28 days) due to low levels of corrosion. Little uptake variation occurred from when a steady state was achieved up to the final sampling point after 100 days. For ease of comparison and viewing, contaminant uptake has been split into several groups, which are detailed as follows:

- Fuel cladding—Mg and Al;
- Fission products—Cs and Sr;
- Lanthanides—Y, La, Ce, Pr, Nd, and Sm;
- Noble metals—Pd, Rh, and Ru.

**Figure 3:**
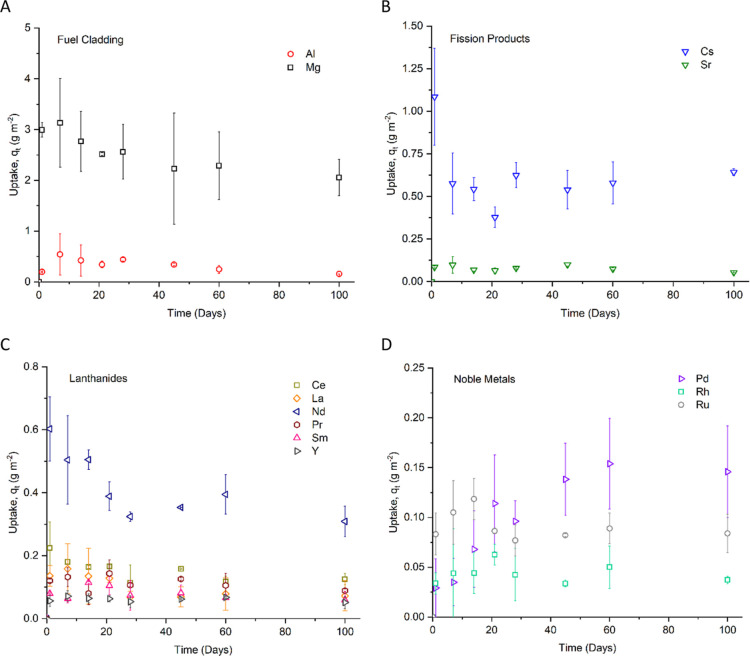
Contaminant uptake profiles for elements present in the Magnox simulant solution (other than Fe, Cr, and Ni) onto 304 stainless steel coupons over the course of 100 days of immersion at 50 °C. (A) Fuel cladding elements; (B) fission products; (C) lanthanides; and (D) noble metals.

Other than Mg and Al, which are principal components of Magnox nuclear reactor fuel cladding, the remaining elements are all produced via nuclear fission. They are grouped based on similarities in properties; that is, Cs and Sr are key medium-lived fission products, the lanthanides all have similar masses (Y is the exception but has similar behavior), and the noble metals are also similar in mass and are highly unreactive. These are shown in Figure 3.

As with other stainless steel contamination studies in nitric acid environments,^12,16,21,27^ an initial peak in uptake was seen for nearly all elements, after between 1 and 14 days, followed by a drop to relatively consistent levels of uptake after between 21 and 28 days. This trend is most clearly seen for Mg, Cs, and Nd. The exception to this was Pd, with its uptake reaching a steady state after approximately 35–40 days.

Species that were added in the highest concentrations, namely, Mg, Cs, and Nd, had the highest levels of uptake at 1.99 ± 0.153, 0.640 ± 0.0177, and 0.310 ± 0.0518 g m^–2^, respectively. Most of the dissolved trivalent contaminant species with initial concentrations of less than 1 g L^–1^ displayed very similar uptake trends, reaching a maximum of approximately 0.2 g m^–2^ or less. Al was the exception, which demonstrated a comparable level of uptake despite its initial higher concentration of 2.4 g L^–1^.

### XPS Analysis

Understanding of the binding mechanisms between the stainless steel surface and the dissolved species can provide a wealth of information.^20,21^ Contamination mechanisms can dictate which decontamination approach may be most appropriate for efficient removal.

As the effects of corrosion were observed to be minimal and solution analysis did not show any significant variations in behavior up to 100 days of exposure, contamination mechanisms were not expected to change over time after steady state was achieved. This is therefore why coupons exposed for 28 days were chosen for detailed analysis, as contamination was difficult to detect using XPS for the more corroded stainless steel coupons (420 days). This was likely due to corrosion effects, reducing measurement resolution. XPS survey scans for the contaminated coupons can be found in the Supporting Information, Figure S1.

**Figure 4:**
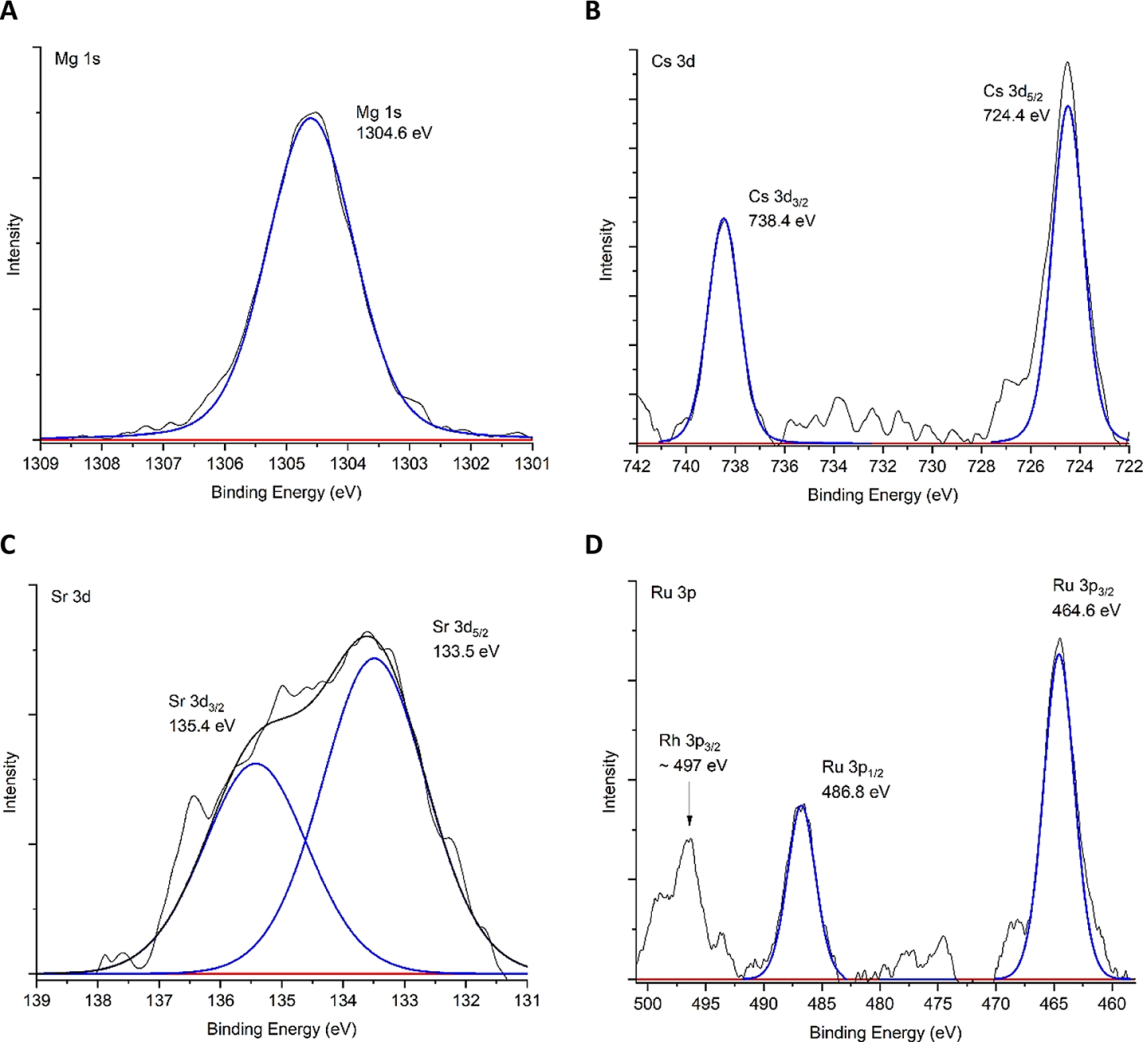
High-resolution XPS spectra of four contaminants present on 304 stainless steel surfaces exposed to the Magnox simulant solution at 50 °C for 28 days. Red —, background; blue —, peak fitting; and black —, cumulative peak fitting. (A) Mg 1s; (B) Cs 3d; (C) Sr 3d; and (D) Ru 3p.

Analysis found that all contaminants present in solution were detected on the surface of the steel. Where possible, peak fitting was employed to determine the most likely speciation of the elements. Four elements (Mg, Cs, Sr, and Ru) were able to be accurately peak-fitted with these presented in Figure 4.

**Figure 5:**
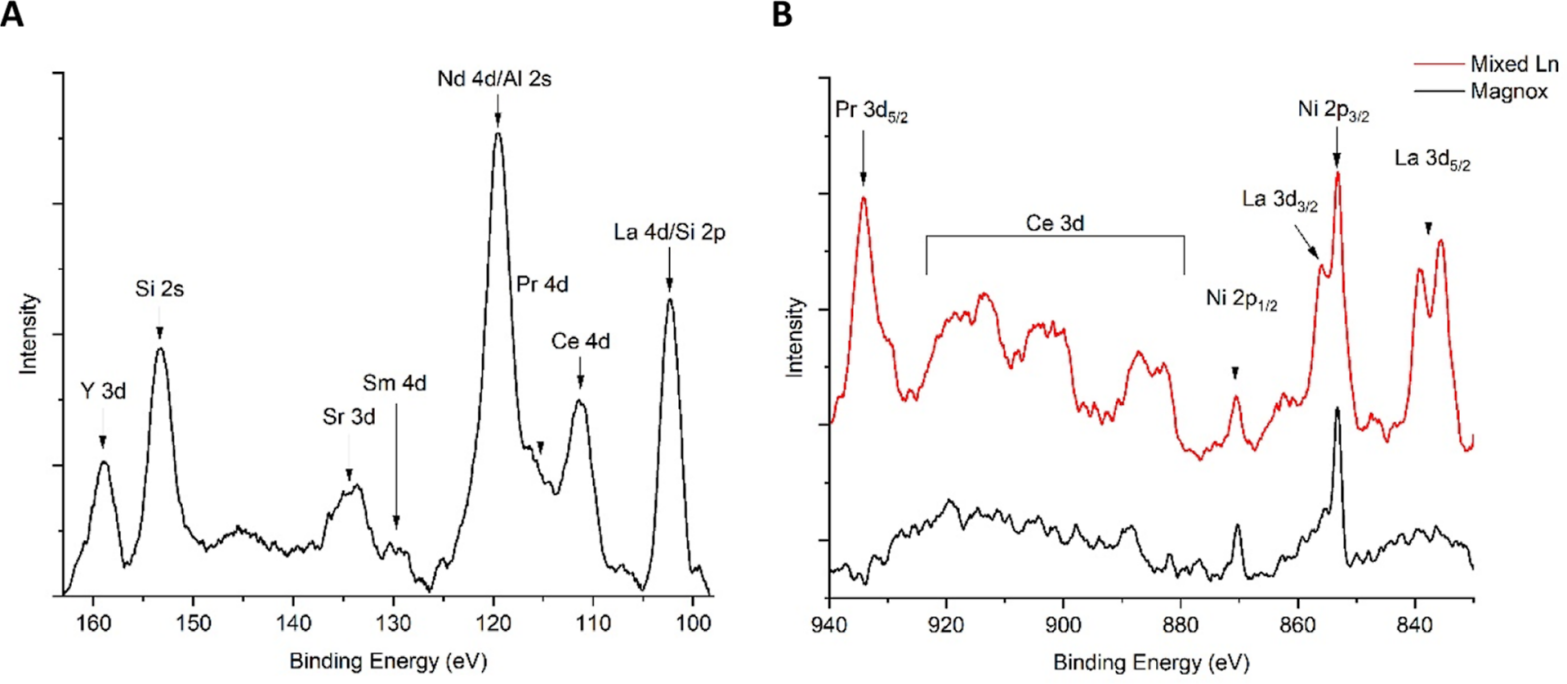
Two XPS regions of interest for lanthanide speciation are present on the 304 stainless steel surfaces after immersion in the Magnox simulant solution at 50 °C for 28 days. (A) Lower energy region predominately identified the 4d orbitals. (B) Comparison of (predominately) 3d orbital signals for a reference-mixed lanthanide (mixed Ln)-contaminated surface (red) and the surface detected for the Magnox-contaminated steel (black).

For the lower energy region between 95 and 165 eV (Figure 5A) covering a wide range of contaminants’ 3d and 4d orbitals, there is evidence that all contaminants of interest in this region were present on the steel surface. For the complex high-energy region between 830 and 940 eV (Figure 5B) which predominantly focuses on lanthanide 3d orbital analysis, a 304 stainless steel coupon contaminated with several lanthanides (contacted with an in-house 1 M HNO_3_ solution with the addition of 1 mM La^3+^, Ce^3+^, Pr^3+^, Nd^3+^, and Sm^3+^ for 28 days) was analyzed with XPS and used as a reference (red) to aid peak identification for the Magnox system (black). Most elements were detected, although peaks were not as clear on the surface of the Magnox-contaminated coupon when compared with the reference.

**Figure 6:**
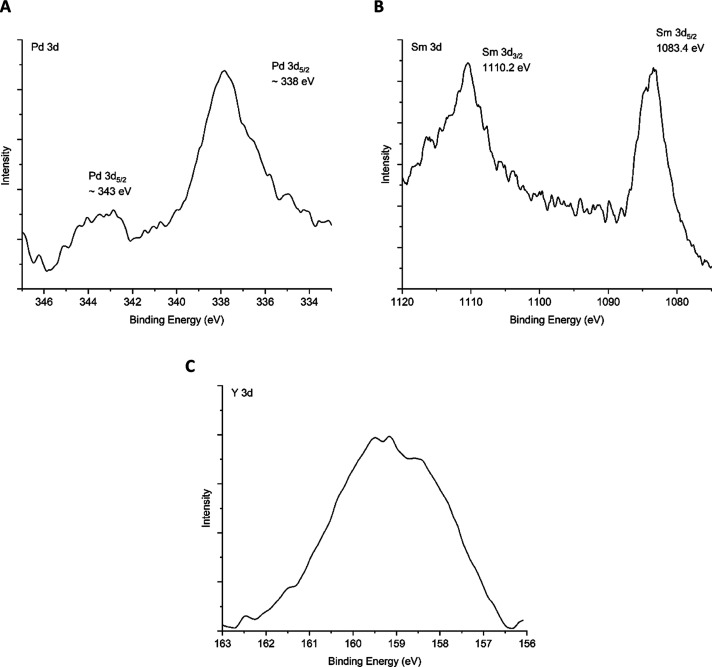
High-resolution XPS spectra for three elements that were present on the 304 stainless steel surface exposed to the Magnox simulant solution at 50 °C for 28 days. (A) Pd 3d; (B) Sm 3d; and (C) Y 3d.

The 3d orbital regions of Pd, Sm, and Y were unable to be peak-fitted due to their irregular peak shape. Coupled with a very small body of XPS data available for comparison for these elements, their most likely speciation was determined based on experimental conditions and trends observed for other elements. The three spectra are provided as evidence of their presence on the surface. These are listed in Figure 6.

### Depth Profiling of Contaminated Stainless Steel

**Figure 7:**
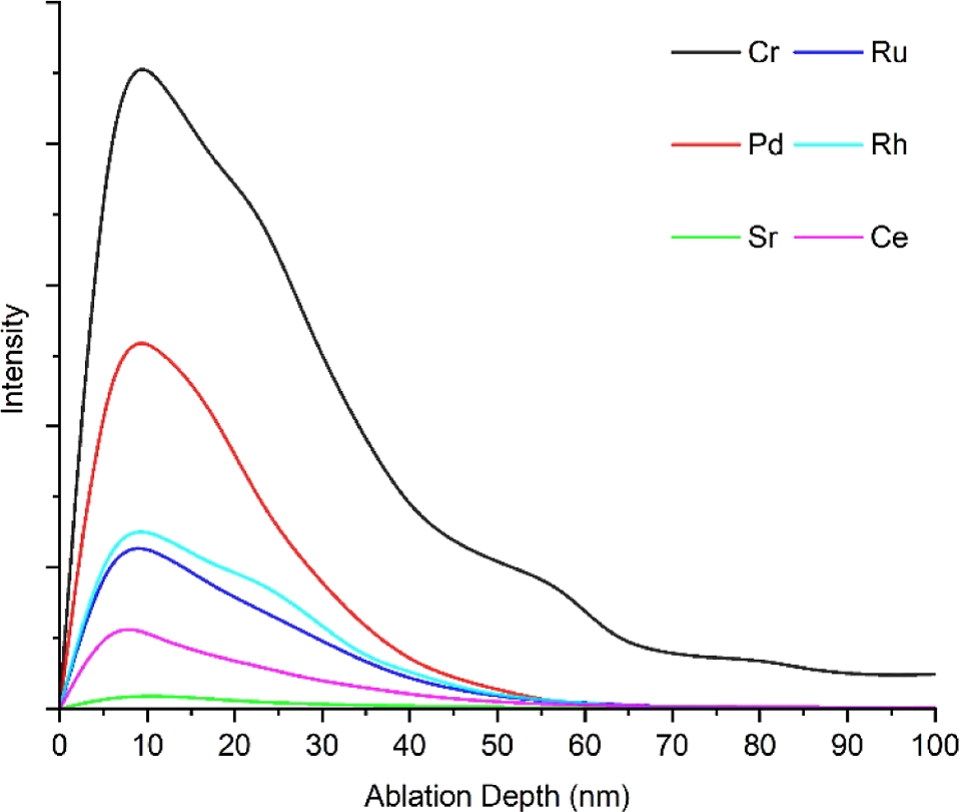
Example depth profile for six elements of interest detected by LA–ICP–MS for a 304 stainless steel coupon immersed in the Magnox simulant solution at 50 °C for 420 days.

An example of a depth profile using LA–ICP–MS (for selected elements) for a coupon exposed to the Magnox simulant solution at 50 °C for 420 days is presented in Figure 7. The average depth of ablation was determined to be 800 nm min^–1^ using white light interferometry (Supporting Information, Figure S2). The average contaminant depths (three random surface points) after both 28 and 420 days are listed in Table 3.

**Table 3:** Average Contaminant Peak Depths within the Stainless Steel from the Magnox Simulant Solution after 28 and 420 Days

element	average peak depth—28 days (nm)	average peak depth—420 days (nm)
Mg	7.40 ± 1.00	9.00 ± 1.73
Al	7.22 ± 1.84	8.79 ± 1.56
Cr	13.5 ± 3.56	11.3 ± 2.10
Sr	6.81 ± 0.196	10.6 ± 0.613
Y	6.76 ± 0.193	9.68 ± 1.03
Ru	6.62 ± 0.311	10.2 ± 1.70
Rh	6.48 ± 0.0351	11.1 ± 1.92
Pd	6.51 ± 0.104	8.76 ± 0.774
Cs	6.83 ± 0.150	8.03 ± 0.645
La	6.89 ± 0.211	9.31 ± 0.151
Ce	6.63 ± 0.0351	9.16 ± 0.560
Pr	6.67 ± 0.0513	8.83 ± 0.499
Nd	6.72 ± 0.116	8.64 ± 0.437
Sm	6.89 ± 0.118	8.77 ± 0.410

After exposure for 28 days, data showed that all elements behaved similarly with no diffusion through the oxide layer. Contamination was observed to be predominantly surface based, between 6 and 8 nm deep, notably lower than the average depth of chromium, a key bulk component of stainless steel. This was not unexpected, as the onset of grain boundary etching was very unlikely within this time frame in the weakly concentrated acidic medium.

In contrast, after 420 days, all elements are diffused slightly further through the oxide layer, closer to the metal–oxide interface (between 8 and 12 nm). Etched grain boundaries and some minor grain dropping may have promoted the diffusion of contaminants to slightly greater depths. Some of the most insoluble species in acidic media (Ru, Rh, and Sr) diffused furthest toward the metal–oxide interface.

## Discussion

### Stainless Steel Corrosion Behavior in MRP Liquors

The increase in the rate of mass loss observed after around 270 days and onward (Figure 1) is attributed to the presence of Fe and Cr corrosion products in solution as a result of the grain boundary etching (seen in Figure 2). This phenomenon has previously been seen to enhance stainless steel corrosion rates.^24,25,28−30^ Very similar mass loss profiles for 304 stainless steel coupons exposed to 12 M HNO_3_ were seen by Barton et al.^21^

However, after further consideration, the very small change in the mass loss rate suggests that dissolved corrosion product concentrations must be extremely small, as the overall mass loss is only 0.25%. Given this, it is therefore likely that the corrosion potential was unaffected and remained near-constant in the passive corrosion domain over the entire immersion period.

**Figure 8:**
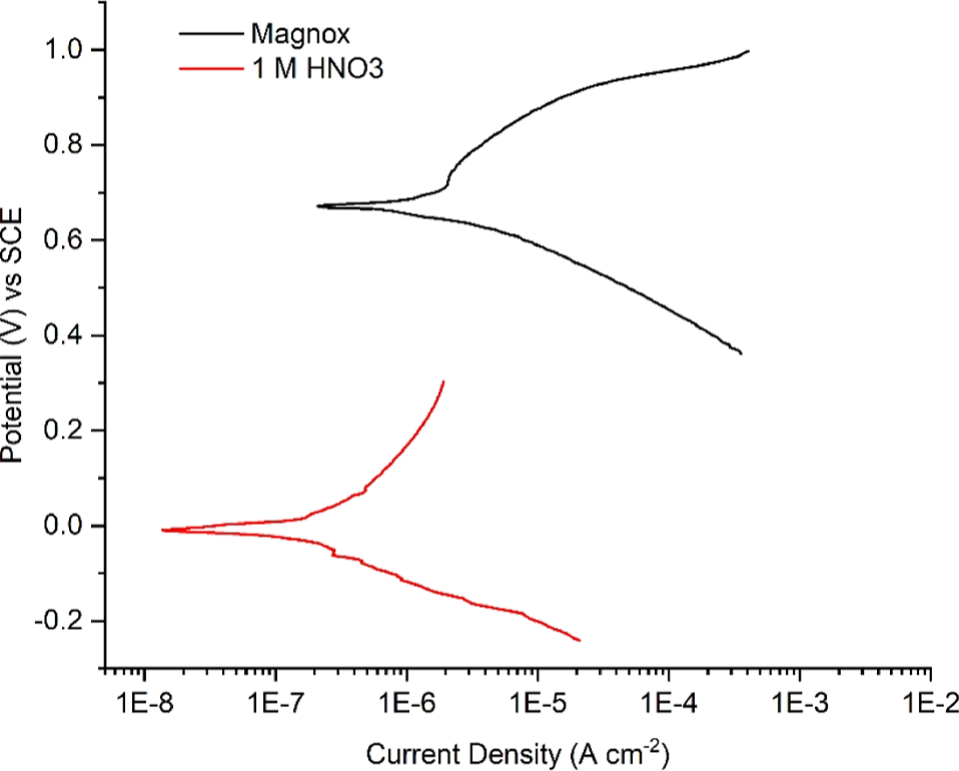
Potentiodynamic polarization curves for 304 stainless steel coupons when exposed to the Magnox simulant solution and 1 M HNO_3_ reference solution. Potential values (V) are presented with respect to the SCE (Hg/HgCl) reference electrode used.

Similarly, previous work by Badet and Poineau showed that the corrosion potential remained within the passive domain for 304L stainless steel held at 45 °C in 8 M HNO_3_ for 6 months, with only minor levels of grain boundary etching observed.^18^ Despite the lower acidity of the system used in this study, comparable levels of grain boundary etching were seen after 270 days. To understand why this occurred, electrochemical potentiodynamic studies were carried out at 50 °C to compare the corrosion potential of the Magnox simulant solution as well as a 1 M HNO_3_ reference solution (without contaminants present). Figure 8 shows a clear increase in the corrosion potential for the Magnox simulant. It also displayed a higher corrosion current density than the control solution which contributes to the higher corrosion rate observed.

The 1 M HNO_3_ reference solution exhibited a corrosion potential of approximately 0 V when it was in contact with the 304 stainless steel coupon. This corrosion potential value suggests that the system sits well within the passive corrosion domain where intergranular corrosion should not occur.^31−33^ The likelihood of grain boundary attack occurring must therefore be strongly influenced by the presence of both an elevated nitrate concentration (due to the high dissolved salt concentration) and redox-active species in solution which were able to elevate the corrosion potential to approximately 0.7 V. The presence of an increased concentration of nitrate in solution has previously been seen to increase corrosion rates, even where no redox-active species were present.^34^ With a greater concentration of NO_3_^–^ present in solution, this can be reduced to NO_2_^–^ in a redox reaction with steel components such as Fe^2+^ and Cr^3+^.

The Magnox liquor has several redox-active species, which could elevate corrosion potential. However, the oxidation states of most of the metallic redox-active ions added to the solution (Cr^3+^, Ru^3+^, and Ce^3+^) are not known to typically increase corrosion rates under these conditions. In strongly acidic media, they may be oxidized at elevated temperatures as has been previously seen,^24,26,35^ but this is unlikely at low acid concentrations. Only the presence of Fe^3+^ could promote corrosion without requiring further oxidation,^29^ as it contributes to the autocatalytic reduction mechanism of nitric acid which has been extensively described.^36−38^ However, it is clear from Figure 1 that the effects of these species on the overall corrosion rate are minimal, suggesting that the extent of oxidation of these ions to higher oxidation states is very small.

Electrochemically determined parameters confirm the low corrosion rates that were expected (Table 4). Despite the elevated corrosion potential of the Magnox solution (673 mV) compared to 1 M HNO_3_ (−13 mV), corrosion currents in both cases are on the nA cm^–2^ scale, resulting in little influence on overall corrosion rates. The electrochemically determined corrosion rate of 3.27 μm year^–1^ was found to be very similar to that of the experimental corrosion rate of 2.97 ± 0.42 μm year^–1^, determined by following the ASTM G31-12a^39^ immersion testing procedure. The similarity of the two corrosion rates determined via alternative approaches suggests that electrochemical corrosion analysis serves as an excellent predictive tool for long-term corrosion in the Magnox simulant liquor.

**Table 4:** Key Parameters Determined from Potentiodynamic Polarization Analysis of the 1 M HNO_3_ and Magnox Simulant Solutions at 50 °C in Contact with 304 Stainless Steel Coupons

solution	*E*_corr_ (mV vs SCE)	*I*_corr_ (nA cm^–2^)	corrosion rate (μm year^–1^)
1 M HNO_3_	–13	187	2.2
Magnox	673	308	3.3

### Long-Term Contaminant Uptake Behavior

Very similar contamination profiles have been seen in recent studies for cesium and strontium contamination of 304 stainless steel coupons in 12 M HNO_3_ at similar temperatures.^12,21^ The initial peak in uptake was followed by a reduction to a steady state after 14–21 days. This was then followed by a further decrease in uptake over time due to corrosion effects and passive layer instability,^21^ a phenomenon not seen in this work. Furthermore, in both studies, strontium uptake exceeded cesium in all cases due to its smaller ionic radius and greater charge density.

However, here, there do not seem to be any obvious contaminant uptake trends with respect to the effects of ionic radius or charge density. This is particularly obvious when comparing the uptake of Mg and Al. The trivalent Al demonstrated uptake more than 10 times smaller than Mg, despite a greater charge density. These findings demonstrate that in simplistic aqueous contaminant systems (i.e., the majority of existing literature in this area), the trends that are observed are unlikely to represent more complex environments, reinforcing the need for studies such as this.

It is now clear that the wide range of parameters influencing contamination (as detailed by Barton et al.^20^) act simultaneously to produce a unique stainless steel contamination profile, which may be difficult to predict. It has been found that, particularly in this low corrosion system, contamination is predominantly dependent on the initial concentration of species in solution and the number of binding sites available, which is then dependent on surface roughness.^17,40^

It could be argued that ions that are mono- and divalent (Cs and Sr) interact preferentially with the surface due to their previously reported contamination mechanism of coprecipitation with corrosion products.^12,21^ However, this would not explain why Nd also demonstrated increased levels of uptake compared to other similar species, especially as it would not coprecipitate with corrosion products, as a trivalent cation. The uptake behavior of Pd was also unexpected, with its uptake peaking at around 35–40 days. It is unclear as to why this occurred, but its behavior may be associated with uptake kinetics.

Therefore, to further elucidate contamination behavior, kinetic uptake behavior was assessed through fitting of the data to the Ho second-order kinetic model.^41^ This was determined to be appropriate given that achieving steady state was generally fast, as it assumes that there are a finite number of sorption sites available for contaminant binding. In addition, similar studies have found that this model is most appropriate for contaminant uptake onto stainless steel surfaces.^12,21^ Both the first-order and Elovich kinetic models were also considered but deemed unsuitable due to the increasingly nonlinear uptake onto the stainless steel. The pseudo-second-order kinetic model can be found in eq 2
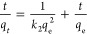
2where *t* is time (days), *q*_*t*_ and *q*_e_ are uptake at time, *t*, and at equilibrium (g m^–2^), respectively, and *k*_2_ is the pseudo-second-order rate constant (m^2^ g^–1^ d^–1^).

Full kinetic plots over 100 days are provided in the Supporting Information, Figure S3, with key parameters provided in Table 5, allowing for comparison of steady-state uptake values.

**Table 5:** Kinetic Parameters Derived from Pseudo-second-Order Kinetic Modeling for Contaminant Uptake onto the 304 Stainless Steel Coupons Immersed in the Magnox Simulant Solution at 50 °C for 100 Days^a^

element	*q*_e_ (g m^–2^)	*k*_2_ (m^2^ g^–1^ d^–1^)	h (g m^–2^ d^–1^)	*r*^2^
Mg	1.99 ± 0.153	0.174 ± 0.149	0.689 ± 0.352	0.995
Cs	0.640 ± 0.0177	0.340 ± 0.262	0.140 ± 0.117	0.971
Nd	0.310 ± 0.0518	0.866 ± 0.410	0.0833 ± 0.0742	0.984
Pd	0.180 ± 0.0347	0.243 ± 0.035	7.89 × 10^–3^ ± 1.23 × 10^–3^	0.911
Al	0.160 ± 2.84 × 10^–3^	0.629 ± 0.180	0.0162 ± 4.04 × 10^–3^	0.921
Ce	0.122 ± 0.0131	2.93 ± 1.73	0.0624 ± 0.0356	0.977
Pr	0.0905 ± 3.96 × 10^–3^	3.46 ± 0.922	0.0284 ± 0.0102	0.975
Ru	0.0816 ± 0.0141	3.79 ± 0.531	0.0732 ± 0.0125	0.996
Sr	0.0570 ± 2.96 × 10^–4^	3.30 ± 1.07	0.0107 ± 3.59 × 10^–3^	0.945
La	0.0540 ± 0.0471	3.29 ± 1.86	9.58 × 10^–3^ ± 0.0451	0.990
Sm	0.0533 ± 0.0240	3.31 ± 1.18	9.40 × 10^–3^ ± 6.54 × 10^–3^	0.969
Y	0.0500 ± 0.0174	5.58 ± 1.11	0.0139 ± 7.97 × 10^–3^	0.966
Rh	0.0383 ± 3.17 × 10^–3^	5.35 ± 0.618	7.85 × 10^–3^ ± 3.86 × 10^–4^	0.968

a Initial sorption rate, h, is determined from the *y* axis intercept.

The kinetic parameters confirm the uptake trends seen in Figure 3, with Mg demonstrating the highest steady-state uptake, *q*_e_, of 1.99 ± 0.153 g m^–2^, followed by Cs (0.640 ± 0.0177 g m^–2^) and Nd (0.310 ± 0.0518 g m^–2^). Trivalent lanthanides tended to achieve equilibrium quickest (as can be seen by larger *k*_2_ values), while elements at the highest concentrations took the longest. Pd is clearly the outlier again, exhibiting an elevated uptake of 0.180 ± 0.0347 g m^–2^, despite an extremely low h value (describing initial sorption rate) of 7.89 × 10^–3^ and a *k*_2_ value comparable to Mg (0.243 vs 0.174), despite its concentration in solution not being at all similar. These unexpected values may be associated with the departure from linearity (*r*^2^ = 0.911), although it is not drastically different from the other species in solution. There are several studies that suggest that the kinetics of palladium adsorption onto ion-exchange resins from acidic (chloride) media closely follow the pseudo-second-order model.^42,43^ Although *k*_2_ values cannot be readily converted for comparison to this work, Pd uptake was seen to be much slower than Rh in a mixed element system (7.5 times slower).^43^ This is further evidence that the kinetics of palladium sorption is extremely slow in a competing system as well as being out-competed for binding sides by the trivalent Rh ion.

## Surface Contamination Speciation

### Divalent Cations and Noble Metals

Incorporation of contaminants into the oxide layer was expected to occur quite readily as passive layer instability associated with high corrosion rates was not present, something that has been seen in strongly acidic conditions.^21^ The most likely forms of contamination were predicted to be metal–oxide interactions (embedded within the oxide layer) and coprecipitation of mono- and divalent species with corrosion products, based nearer to the outermost surface.^10,12^ Most likely, speciation for each element is discussed and presented in Table 6.

**Table 6:** Most Likely Binding Energies and Speciations for the Elements Detected on the 304 Stainless Steel Surface after Immersion in the Magnox Simulant Solution at 50 °C for 28 Days

component	binding energy (eV)	most likely speciation	refs
Mg 1s	1304.6	MgO	(44 and 45)
Cs 3d_5/2_, Cs 3d_3/2_	724.4, 738.4	Cs_2_CrO_4_/Cs_2_Cr_2_O_7_	(46)
Sr 3d_5/2_, Sr 3d_3/2_	133.5, 135.4	SrCrO_4_	(12 and 48)
Ru 3p_3/2_, Ru 3p_1/2_	464.6, 486.8	RuO_2_	(49−51)
Rh 3p_3/2_	∼497	Rh_2_O_3_	(54)
La 3d_5/2_	835.4, 839.2 (doublet)	La_2_O_3_	(57)
Ce 3d_5/2_, Ce 3d_3/2_ (Ce^3+^)	882.6, 899.8	Ce_2_O_3_	(58)
Ce 3d_5/2_, Ce 3d_3/2_ (Ce^4+^)	887.0, 904.1	CeO_2_	(62 and 63)
Pr 3d_5/2_	934.0	Pr_2_O_3_	(66−68)
Pd 3d_5/2_, Pd 3d_3/2_	∼338, ∼43	PdO	(69)
Sm 3d_5/2_, Sm 3d_3/2_	1083.4, 1110.2	Sm_2_O_3_	(75−77)
Y 3d	157–162	Y_2_O_3_	(78 and 79)

Analysis of the Mg 1s binding energy region (Figure 4A) found a clear, high-intensity peak associated with its high uptake value determined through solution analysis (1.99 ± 0.153 g m^–2^). The binding energy was determined to be 1304.6 eV, corresponding to a Mg–O bonding environment. Literature analysis alludes to a most likely speciation of MgO based on reported binding energies for other inorganic systems.^44,45^

Within the Cs 3d region (Figure 4B), Cs 3d_5/2_ and Cs 3d_3/2_ were present with values of 724.4 and 738.4 eV, respectively. The most likely bonding environment was identified to be Cs_2_CrO_4_.^46^ This varied from previous literature, as a similar study testing cesium contamination of 304 stainless steel in 12 M HNO_3_ at 60 °C identified Cs_2_Cr_2_O_7_ as the most probable species at the surface.^12^ However, Ningshen et al. identified using Raman spectroscopy that mixed Cr(III)/Cr(VI) oxides were present on stainless steel exposed to 5 M HNO_3_ at a similar temperature (70 °C), suggesting that both chromate and dichromate species could be present.^47^

Similar to the Cs 3d bonding environment, the Sr 3d_5/2_ and Sr 3d_3/2_ binding energy values of 133.5 and 135.4 eV identified in Figure 4C correspond to SrCrO_4_. This is in agreement with previous steel contamination work.^12,27,48^ These findings confirm the hypothesis that the larger mono- and divalent cations co-precipitate preferentially with chromium.

Finally, the Ru 3p photoelectron lines of Ru 3p_3/2_ and Ru 3p_1/2_ had values of 464.6 and 486.8 eV, respectively (Figure 4D). Compared with previous literature, these values align strongly with the presence of Ru^4+^ ions and RuO_2_.^49−51^ This was unexpected as ruthenium was initially added in its Ru^3+^ form as ruthenium nitrosyl nitrate, suggesting that it was oxidized in solution to its higher +4 oxidation state. Ruthenium is known to behave in a complex manner; therefore, it is not necessarily unexpected in the acidic medium.^52^ In addition, within the Ru 3p region of interest is the Rh 3p_3/2_ line, which clearly identifies the presence of rhodium on the steel surface. The preferred Rh 3d orbital (which is typically referred to in the literature) is overlapped by the Mg KLL Auger peak, and there is little to no literature analyzing the Rh 3p orbital, making assessment difficult. Considering ruthenium’s behavior, as a noble metal, rhodium may have been expected to behave similarly, although oxidation of Rh^3+^ to Rh^4+^ appears highly unlikely due to the need for an extremely oxidizing environment.^53^ As identified previously, boiling of HNO_3_ solutions is generally required to readily oxidize species in solution;^24,25,30^ relatively low temperatures such as those used in recent studies have low oxidizing power.^12,21,27^ With this in mind, the most likely species of Rh would therefore be Rh_2_O_3_,^54^ as RhO_2_ is typically found as a volatile gaseous product in off-gas streams^55^ rather than in aqueous nuclear environments.

### Lanthanides

Analysis of the two broad regions identified a wide range of contaminants present on the steel surfaces after exposure to the Magnox liquor. Initially focusing on the lower energy region (95–165 eV) seen in Figure 5A, overlapping of La 4d and Si 2p, along with Nd 4d and Al 2s orbitals, posed issues for identification. There are no clear alternatives for Al, as the Al 2p orbital also has numerous overlaps at ∼74 eV. However, it is likely that Al_2_O_3_ is formed given the interactions of other trivalent cations with the passive layer.

There was evidence of all other lanthanides present on the surface (Ce, Pr, Sm, and Y), but fitting was challenging due to peak overlapping. Furthermore, given that the spin–orbit splitting doublet separation is extremely small for the 4d orbitals,^56^ the 3d orbitals at higher binding energies (800–900 eV) were identified as appropriate alternatives for analysis due to larger doublet separations (>10 eV).

Uptake of the lanthanide species onto the 304 stainless steel coupons in the reference solution is considerably greater than in the Magnox simulant, as observed for the 830–940 eV region (Figure 5B). This reference system serves as a basis for understanding lanthanide speciation on the stainless steel surfaces in a multi-element system, as speciation is unlikely to change in similar acidic media. It has also been identified that other elements preferentially interact with the steel from the Magnox simulant liquor, resulting in lower levels of uptake for the lanthanides, despite much higher initial concentrations in the simulant compared with the reference. For the coupon exposed to the Magnox simulant, La and Ce concentrations must be approaching their limits of detection, while Pr is seemingly absent at its expected binding energy.

Analysis of the expected binding energies for La reveals approximate values of 835.4 and 839.2 eV for La 3d_5/2_, which is the most appropriate, as the La 3d_3/2_ orbital overlaps with the Ni 2p_3/2_ line at ∼855 eV. These binding energies correspond to a most likely speciation of La_2_O_3_.^57^ The complex Ce 3d region is challenging to deconvolute, with six peaks associated with cerium species. When analyzing existing literature, values of 882.6 and 899.8 eV tended to be associated with Ce^3+^ species;^58^ however, a number of studies also found values of 882.6 eV for Ce^4+^ species, albeit with its doublet at ∼898.5.^59−61^ Studies point to Ce^3+^ for 887.0 and 904.1 eV for Ce 3d_5/2_ and Ce 3d_3/2_, respectively.^62,63^ A value of ∼917.0 eV is typically assigned to Ce^4+^; therefore, it is believed that the values around 913.0 and 919.0 eV are satellite peaks which may hide its presence.^64,65^

Pr 3d_5/2_ is the final electron orbital analyzed in this region. A value of 934.0 eV corresponds to a most likely species of Pr_2_O_3_,^66−68^ which is logical due to assignments of La_2_O_3_ and Ce_2_O_3_ for the other lanthanides. The Nd 3d region is omitted due to its overlap with the O KLL Auger peak but would be expected to follow the same speciation trend.

### Remaining Species of Interest

For the three remaining species of interest, namely, Pd, Sm, and Y, justification for their most likely speciation is discussed here. Their spectra are presented in Figure 6.

The Pd 3d region has a strong Pd 3d_5/2_ signal at ∼338 eV, yet the intensity of the Pd 3d_3/2_ peak at ∼343 eV is much smaller than expected (Figure 6A). It is not clear as to why the intensity of Pd 3d_5/2_ is considerably greater, especially as there do not appear to be Auger emissions in this region or any interferences. Based on existing literature, it appears that the shape of Pd 3d_5/2_ may correspond to the presence of both divalent and tetravalent Pd in oxide form.^69^ However, the presence of a higher oxidation state is very unlikely, requiring an oxidizing potential upward of 1 V.^70^ The additional species may therefore be associated with hydrolysis products of Pd in the acidic media, most probably Pd(OH)_2_.^71,72^ Hydrolysis in acidic conditions has also been seen for other species that would be present in reprocessing liquors, including U and Pu, forming colloidal species at pH values as low as 0.^73,74^

Peak values for Sm 3d_5/2_ and Sm 3d_3/2_ at approximately 1083.4 and 1110.2 eV are widely accepted to be associated with Sm_2_O_3_ (Figure 6B).^75−77^ It is interesting to note that even though samarium was initially added to the Magnox simulant in oxide salt form, it appears to have remained unchanged when interacting with the steel. Furthermore, its speciation would also be expected to follow the trend of the other lanthanides, which are incorporated as a trivalent metal oxide.

Finally, the Y 3d orbital shows a binding energy profile very similar to that of the Sr 3d orbital (Figure 6C). However, previous literature has shown that Y 3d profiles with this shape correspond to multiple yttrium species.^78,79^ It would, however, be expected that Y follows the trend of the lanthanides given it is a trivalent cation, forming Y_2_O_3_. The second species present is unknown, but previous work has alluded to the presence of Y(OH)_3_.^79^ This would however be unexpected in acidic media as hydrolysis of yttrium and rare-earth elements occurs in alkaline conditions.^80^

XPS analysis has proven to be an excellent tool for element identification even for this complex system. Speciation for elements initially added in the nitrate form has been determined with the majority being incorporated into the steel’s oxide layer in metal oxide form as expected. Some exceptions have been identified, with cesium and strontium incorporated via a coprecipitation mechanism with chromium. In addition, ruthenium appears to be incorporated as an oxide with an elevated oxidation state (Ru^4+^) compared to its initial form. This finding is important as Ru oxides are notoriously insoluble in water, with previous work finding noble metals bind tenaciously to the steel oxide layer and are difficult to remove via traditional decontamination approaches.^81^ Furthermore, the presence of Ce^4+^ also suggests oxidation from Ce^3+^. This phenomenon, plus the oxidation of Ru^3+^, may confirm the previously discussed hypothesis regarding the elevated corrosion potential of the system.

### Assessment of Contaminant Distribution with Increasing Depth

Depth profiling is a key characterization tool for determining whether contaminant diffusion beyond the oxide layer and into the bulk substrate has occurred, which would have significant implications regarding nuclear plant decontamination and decommissioning planning. Previous work has utilized GD-OES and PP-time-of-flight (ToF)-MS successfully for the depth profiling of 304 stainless steel contaminated with cesium, strontium, and a range of lanthanides individually.^12,15^ However, for increasingly complex systems, neither technique remains suitable due to their inability to maintain a vacuum seal with severely corroded coupons, making analysis impossible. Not only that, GD-OES is also unable to measure multiple “atypical” elements simultaneously, making it a poor candidate for depth profiling of samples that have been exposed to complex environments.

The adoption of LA–ICP–MS for stainless steel depth profiling allowed for improved detection limits, low ablation rates, and tunability, while overcoming the challenges associated with the aforementioned techniques. Barton et al. successfully employed this technique previously for severely corroded 304 stainless steel coupons that had been exposed to 12 M HNO_3_ for 420 days at 50 °C.^21^ This depth profiling approach was therefore deemed to be a suitable option for analysis.

Contaminants were predominantly situated near the immediate surface of the 304 stainless steel coupons, with a contaminant rich area of the oxide layer located between 6 and 8 nm after 28 days and 8 and 12 nm after 420 days. Species associated with similar blocks of the periodic table, such as lanthanides and noble metals, displayed very similar depths of contamination at both analysis points. This behavior is believed to be due to the similar ionic radii and isotopic masses of these elements.

**Figure 9:**
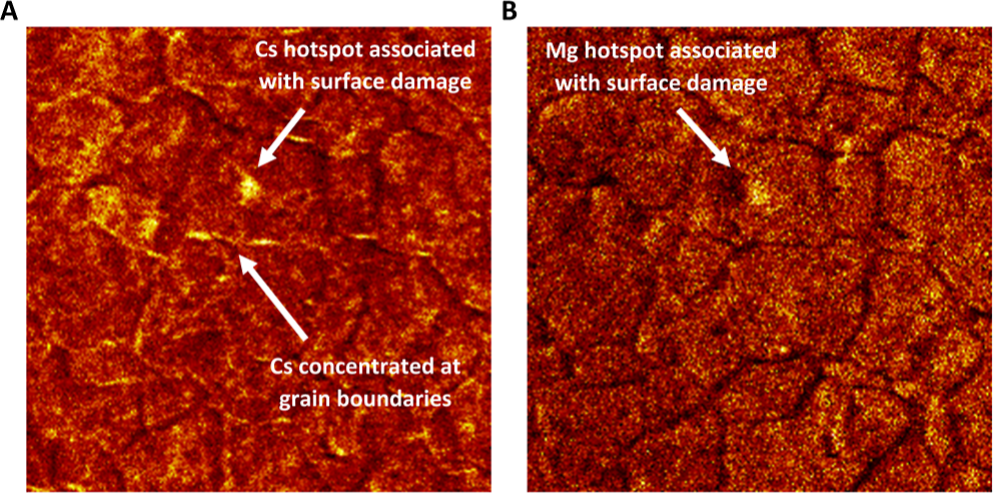
ToF-SIMS maps of Cs (A) and Mg (B) contamination on a 304 stainless steel coupon exposed to the Magnox simulant for 420 days at 50 °C.

Cesium was seen to be the most surface-based contaminant (accounting for error assessment), with the lowest average depth of all elements at 8.03 ± 0.645 nm. This finding is confirmed by time-of-flight secondary ion mass spectrometry (ToF-SIMS), with widespread distribution of cesium seen across the surface, as well as a couple of contamination hotspots, suggesting that it is indeed a predominantly surface-based contaminant (Figure 9A). This is in agreement with previous ToF-SIMS observations by Lang et al.^12^ Magnesium was also found to be evenly distributed across the surface, as was expected due to its high level of uptake (Figure 9B).

**Figure 10:**
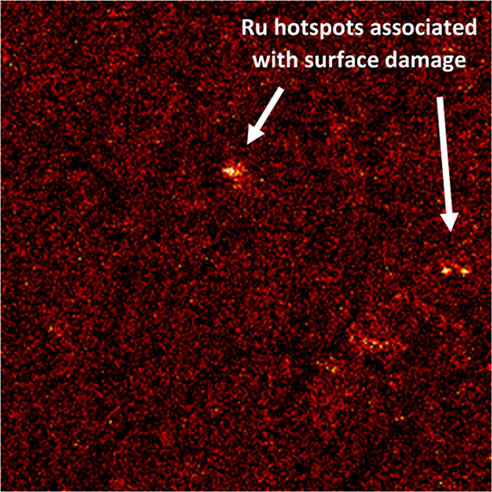
ToF-SIMS map of Ru contamination on a 304 stainless steel coupon exposed to the Magnox simulant for 420 days at 50 °C.

Ruthenium was the only element other than Cs and Mg that was clearly concentrated at specific locations within the sampling area (Figure 10). It appears that the hotspot is situated within an area of surface damage or a crevice. The increased level of contaminant uptake here suggests that surface roughness is considerably higher, creating more binding sites available for interaction with the abundance of cationic species.^40^ The remaining elements were distributed across the surface at relatively low concentrations, correlating with solution uptake analysis. ToF-SIMS imaging for these species can be found in the Supporting Information, Figure S4.

Variations in oxide layer thickness over time are also of interest alongside contamination depth; to ensure complete decontamination of the material, the entire oxide layer may need to be removed. Across an entire reprocessing facility, a large increase in the oxide layer thickness may result in significant volumes of additional waste being generated.

After averaging the oxide layer thicknesses determined through depth profiling at three randomly chosen points on the 304 stainless steel coupons, the average oxide thickness for coupons exposed to the Magnox simulant liquor for 28 days was determined to be 57.2 ± 10.5 nm, whereas after 420 days, it was found to be 84.7 ± 3.75 nm. Metal–oxide interface depths were found to be relatively consistent, with no evidence of contamination beyond this depth for any coupons at either exposure time. It is believed that the increase in oxide layer thickness over time is associated with the incorporation of cations into the oxide layer over time (such as those with slow uptake/diffusion kinetics like Pd), plus the increased concentration of dissolved oxygen diffusing inward, as a result of the high NO_3_^–^ concentration added initially.

A similar work by Kerry using Focused Ion Beam found oxide layer thicknesses of approximately 70 nm in 4 M HNO_3_, and up to 110 nm in 12 M HNO_3_, for 304L stainless steel coupons exposed for 30 days at 50 °C with the presence of Eu^3+^.^82^ Furthermore, an interesting comparison in the same study was the average oxide layer thickness after 7 and 30 days. After 7 days, it was determined as 27 ± 12 nm, while after 30 days, 67 ± 38 nm.^82^

The findings of this study are confirmation that oxide layer thicknesses are greater in acidic media with increasing contact time, as previous work identified.^82^ This is in contrast to studies that examine oxide layer thickness (typically after extremely short time periods) in nitric acid media, which report oxide layers of 10 nm or less,^31,83^ and those in high-temperature oxidizing systems where the oxide layer is on the order of a few microns thick.^74,84^ Determination of the oxide layer thickness after long periods of exposure is key to the development of appropriate decontamination approaches, which will require the removal of this layer to achieve high decontamination factors.

## Conclusions

The development of a characterization portfolio consisting of surface-based and depth profiling techniques to comprehensively detail contamination and corrosion of 304 stainless steel in a simulant Magnox reprocessing liquor has been successfully achieved. It is one of very few studies to examine the contamination and corrosion behavior of stainless steel for extended time periods at elevated temperatures. Corrosion in the dilute acidic media was limited to surface attack, despite high concentrations of both nitrate and oxidizing ions which elevated the corrosion potential to approximately +700 mV (vs SCE). With passivity maintained, corrosion rates were measured to be extremely low at approximately 3 μm year^–1^, confirmed with electrochemical analysis.

Contaminant uptake over 100 days was dominated by those elements present in the highest concentrations in solution, notably Mg, Cs, and Nd. This was confirmed by ToF-SIMS mapping. Other elements present in very similar initial concentrations demonstrated near-identical uptakes (∼0.1 g m^–2^). XPS analysis found that most cations interacted with the steel to form metal oxides, other than Cs and Sr, which formed chromates (Cs_2_CrO_4_/Cs_2_Cr_2_O_7_ and SrCrO_4_) via a co-precipitation mechanism. Ru and Ce were present with elevated oxidation states (Ru^4+^ and Ce^4+^) due to the acidic media promoting the oxidation of the cations initially present. Depth profiling using LA–ICP–MS identified that contamination did not diffuse into the bulk substrate after 28 days or 420 days. Average depth of contamination had increased by a few nanometers but was attributed to the increase in oxide layer thickness over time, from 57 to 84 nm.

This is one of the first studies to provide detailed characterization of how a simulant solution interacts with stainless steel under industrially relevant conditions for extended periods. This work can be used as a basis for predictive modeling of long-term steel contamination for existing and future nuclear reprocessing facilities, as well as future laboratory simulations which can enhance the understanding of aqueous nuclear contamination scenarios. The findings of this work, particularly the mechanisms and depth of contamination, are hoped to inform the development or application of decontamination techniques which will lead to waste volume minimization, subsequently reducing associated costs and future environmental burden.

## Supporting Information Available

XPS wide scans, white light interferometry crater depth analysis, pseudo-second-order kinetic modeling plots, and ToF-SIMS mapping (PDF)
