# Supplementary material for: Development of a Multi-technique Characterization Portfolio for Stainless Steels Exposed to Magnox Reprocessing Liquors
Source: ACS Omega. 2023 Nov 17;8(48):46151–64. doi: 10.1021/acsomega.3c07240 (PMC10702187; doi:10.1021/acsomega.3c07240)
Supplement: Supplementary file 1 — ao3c07240_si_001.pdf [file ao3c07240_si_001.pdf]

Supporting Information

Development of a Multi-Technique Characterisation Portfolio for Stainless Steels Exposed to Magnox Reprocessing Liquors

Daniel N. T. Barton[1*], Anna E. Denman[2], Tatiana Grebennikova[1], Thomas Carey[3], Dirk L. Engelberg[4], Clint A. Sharrad[1]

[1]Department of Chemical Engineering, The University of Manchester, Oxford Road, Manchester, M13 9PL, United Kingdom

[2]Department of Earth and Environmental Sciences, The University of Manchester, Oxford Road, Manchester, M13 9PL, United Kingdom

[3]National Nuclear Laboratory, Chadwick House, Warrington, WA3 6AE

[4]Department of Materials, The University of Manchester, Oxford Road, Manchester, M13 9PL, United Kingdom

* Corresponding author

Figure S1

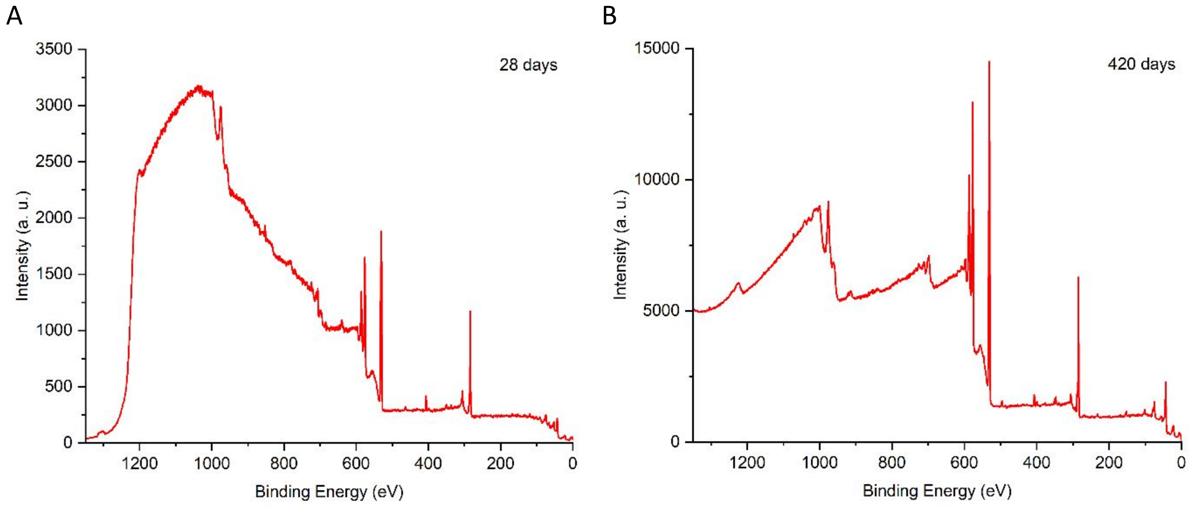

Figure S1. XPS widescans for stainless steel coupons exposed to Magnox simulant solutions for 28 days **(A)** and 420 days **(B).**

Figure S2

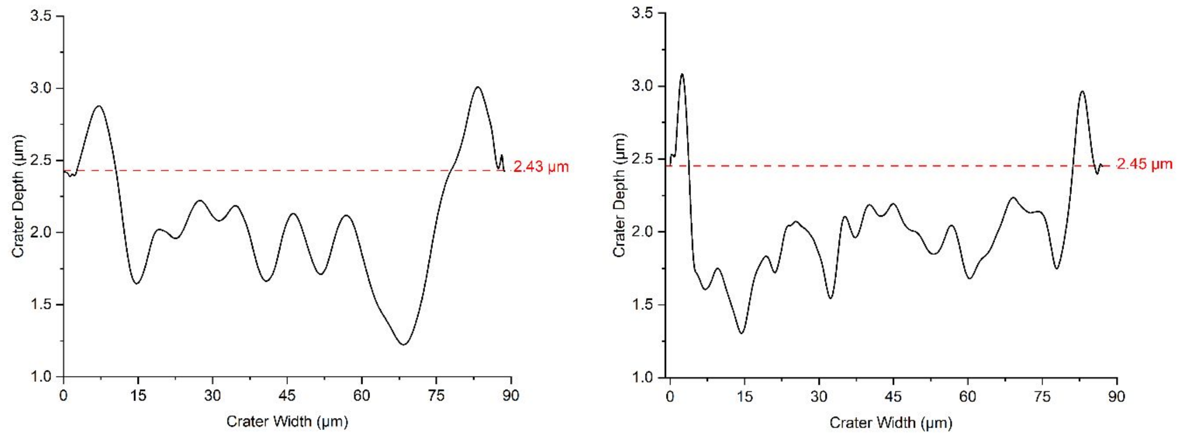

Figure S2. White light interferometry analysis of the craters formed during laser ablation depth profiling. An average depth of 800 nm min^{-1} was calculated based on the average depth of ablation being ~560 nm after 40 seconds, the equivalent of 200 shots at 1 J cm^{-2} and 5 Hz repetition rate.

Figure S3

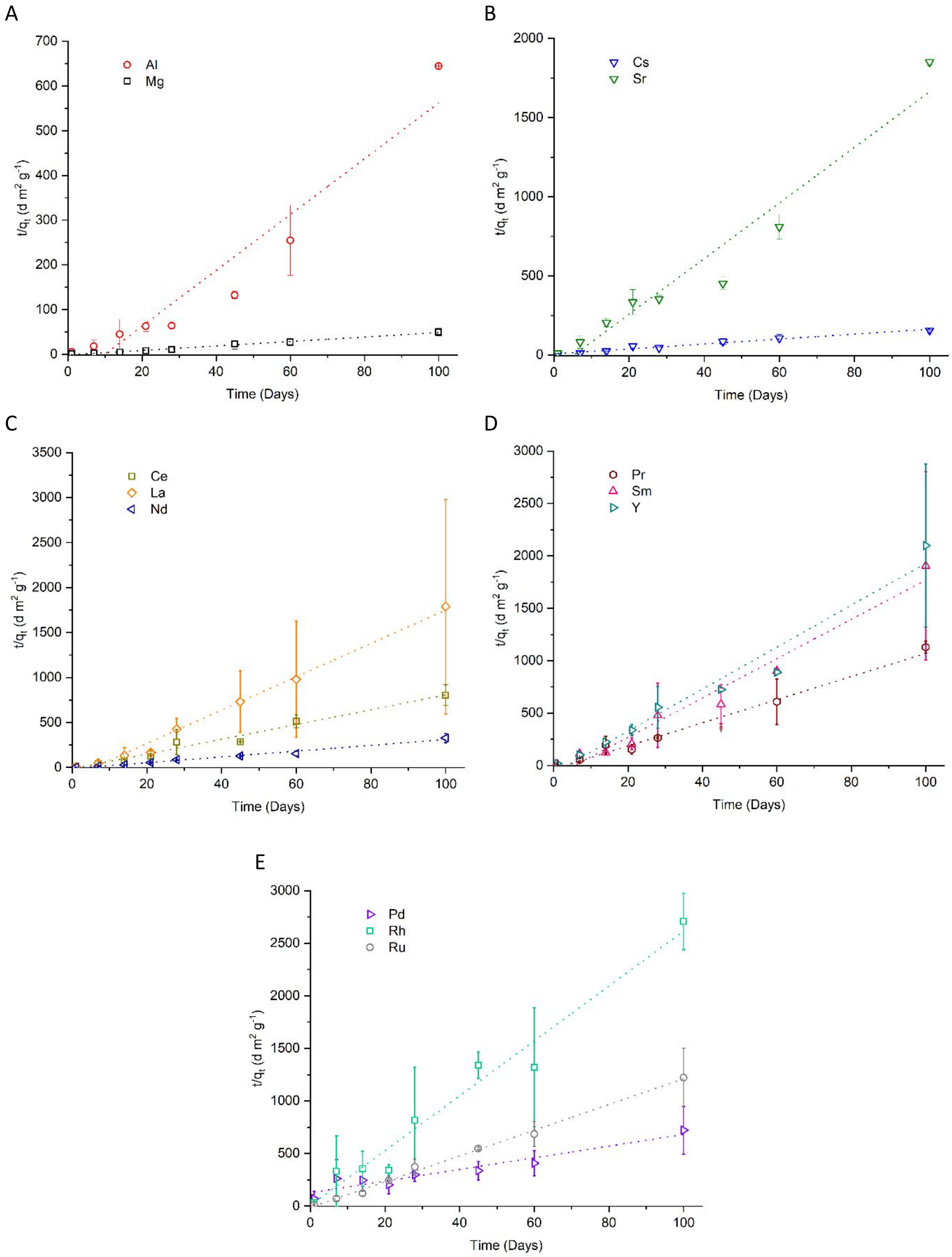

Figure S3. Pseudo-second order kinetic modelling plots over the course of 100 days for uptake of elements from the Magnox simulant solution on to stainless steel coupons. **A**: Fuel Cladding elements; **B**; Fission products; **C**: Lanthanides 1; **D**: Lanthanides 2; **E**: Noble metals.

Figure S4

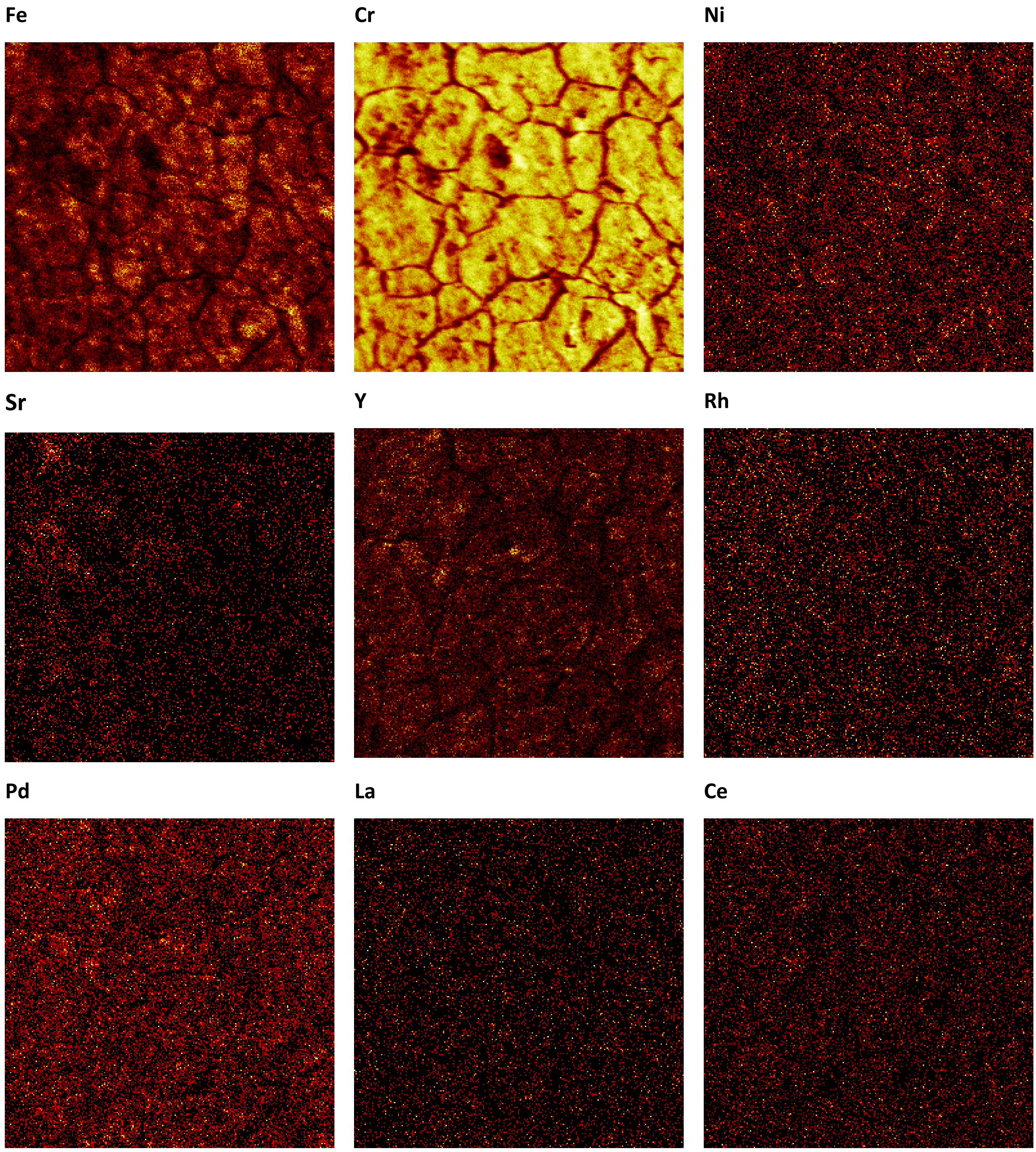

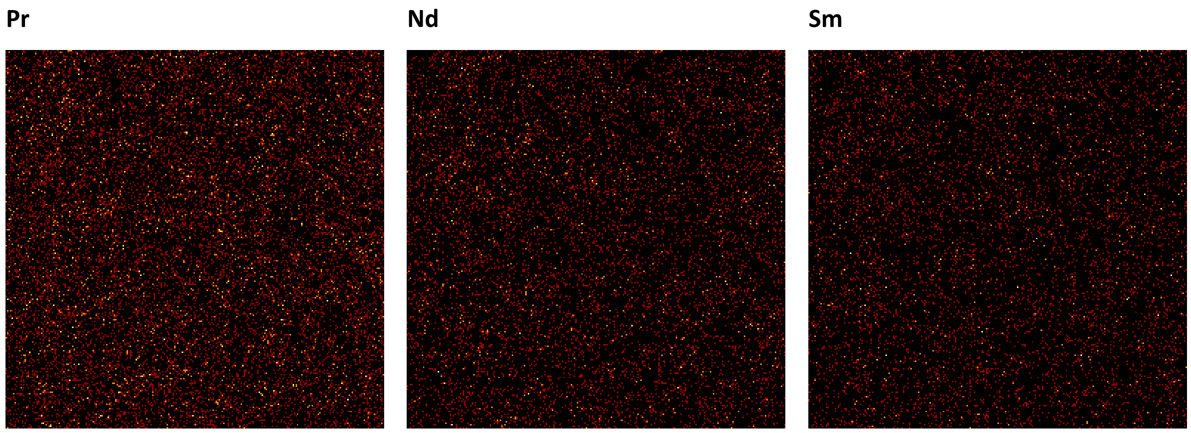

Figure S4. Time-of-Flight Secondary Ion Mass Spectrometry maps for a 100 x 100 µm area on the 304 stainless steel coupons exposed to the Magnox simulant for 420 days.
